# Pathology and Clinics of Naturally Occurring Low-Virulence Variants of African Swine Fever Emerged in Domestic Pigs in the South Caucasus

**DOI:** 10.3390/pathogens13020130

**Published:** 2024-01-29

**Authors:** Hranush Avagyan, Sona Hakobyan, Bagrat Baghdasaryan, Hranush Arzumanyan, Arpine Poghosyan, Nane Bayramyan, Anna Semerjyan, Mariam Sargsyan, Henry Voskanyan, Tigranuhi Vardanyan, Naira Karalyan, Lina Hakobyan, Liana Abroyan, Aida Avetisyan, Elena Karalova, Zara Semerjyan, Zaven Karalyan

**Affiliations:** 1 Laboratory of Cell Biology and Virology, Institute of Molecular Biology of NAS RA, Yerevan 0014, Armenia; a.avagian@yahoo.com (H.A.); 777sona7@gmail.com (S.H.); artdrbaghdasaryan@gmail.com (B.B.); arzumanyan0712@gmail.com (H.A.); arpi.poghosyan21@gmail.com (A.P.); naneramaz@mail.ru (N.B.); aleksvoskanyan@mail.ru (H.V.); tika.vardanyan86@mail.ru (T.V.); lina.hakobyan@gmail.com (L.H.); abroyan.liana@gmail.com (L.A.); a.avetis@mail.ru (A.A.); hatussili@yahoo.com (E.K.); zarasem@gmail.com (Z.S.); 2 Experimental Laboratory, Yerevan State Medical University after M. Heratsy, Yerevan 0025, Armenia; 3 Department of Medical Biology and Genetics, Yerevan State Medical University after M. Heratsy, Yerevan 0025, Armenia; annasemerjian@hotmail.com; 4 Department of Epizootiology and Parasitology, Armenian National Agrarian University, Yerevan 0009, Armenia; mariam.sargsyan.1960@mail.ru; 5 Department of Pathological Anatomy and Clinical Morphology, Yerevan State Medical University after M. Heratsi, Yerevan 0025, Armenia; naira_karalyan@yahoo.com

**Keywords:** African swine fever virus, chronization, immunopathology, histopathology, clinics

## Abstract

Shortly after the establishment of African swine fever virus (ASFV) genotype II in 2007, cases of acute fatal infection were observed. However, after several years of circulation in the Eurasian region, the clinical signs of the disease changed. Currently, this disease can occur acutely, subclinically, chronically, or asymptomatically. Cases of the complete recovery of infected pigs, and the disappearance of ASFV from their tissues and secretions have been described. This form of the disease first appeared in Armenia at the end of 2011. This virus was described and identified as the Dilijan2011IMB strain. The goal of our research was to study the main features of clinical, pathological, immunological, virological, and genetic parameters involved in the development of new forms of African swine fever (ASF). Chronic ASF was characterized with low titers of the virus and a decrease in the intensity of hemadsorption. Additionally, a reduced intensity in clinical symptoms and pathoanatomical results was noted. The absolute, but not the relative number of immune cells changes; the neutropenia (in bone marrow and spleen), lymphopenia (in bone marrow), lymphocytosis (only in spleen), lymphoid cell depletion (in bone marrow), and pancytopenia (in bone marrow) observed in the chronic form of ASF were less pronounced compared to in the acute form. When comparing the late stage of chronic ASF to the acute form, the key cytological indicators in the spleen, lymph nodes, and blood were less severe in the chronic stage. Bone marrow failure in the chronic form, expressed in a pronounced decrease in all cell types, generally coincided with the data in the acute form of ASF. The same data were obtained after assessing serum TNF-alpha levels. Thus, we can conclude that the chronic form of ASF occurs due to a less pronounced immune response, as well as a decrease in virus titers in the blood and tissues of infected pigs.

## 1. Introduction

African swine fever virus (ASFV) (genotype II) entered Eurasia in 2007, and at the initial stage of circulation, showed the presence of acute and super-acute forms of the disease with a 100% lethal outcome [1,2,3]. However, after several years, individual cases of subacute forms of the disease have been recorded. Thus, at the end of 2011, cases of chronic or subacute form of ASF were identified in Dilijan (Armenia) [4].

As African swine fever (ASF) spreads beyond its initial African location to domestic pigs in other regions, the disease’s progression typically accelerates. This results in more animals entering subacute and chronic phases, potentially leading to longer lifespans in affected pigs and reduced visible clinical symptoms. Thus, in Spain, two years after the introduction of ASF, domestic virus-carrying pigs were detected [5]. Some studies have identified reduced-virulence isolate ASFV genomes in apparently healthy pigs in Uganda and Kenya [6,7]. In Armenia, similar processes occurred 4 years after the emergence of ASFV.

Previously, the following main characteristics of the chronicity of the disease were recorded: (1) a reduction of the virus in infected organs and blood; (2) a decrease in the hemadsorption activity of the virus; (3) a decrease in the manifestation of the main clinical and laboratory parameters of ASF (as decreased in hemorrhages and immunopathological manifestations); and (4) prolonged viremia [4].

In 2018–2020, several outbreaks of an unknown disease occurred in the South Caucasus in pigs vaccinated against classical swine fever. After field and laboratory examinations, the presence of the African swine fever virus in all the studied samples was confirmed in our laboratory.

In recent years, there have been increasing reports of new cases of ASF virus with an atypical course. The pathology is characterized by a significantly prolonged disease duration, sometimes reduced mortality, and prolonged viremia. Such changes are often associated with the circulation of the virus in the feral pig population [8,9]. In China, there have been many cases of ASF virus with reduced virulence in domestic pigs since 2018 [10,11].

Attenuated viruses cause less severe pathological alterations compared to the more aggressive pathological changes induced by their virulent wild-type origins. Although there is evidence of the presence of mildly pathogenic strains of ASFV belonging to genotype II [10,12,13,14,15], no study has provided a definitive description of the pathology development in natural attenuation. The current information on the hemadsorption activity (Leitão et al., 2001) prospects of decreasing activity usually correlate with attenuation and prospects of their use as vaccines [15,16], so it has been shown that naturally attenuated isolates can provide fairly reliable protection in wild boar [17]. In this article, we try to show the differences in the pathogenetic characteristics of naturally attenuated strains of ASFV detected in the South Caucasus in 2018–2020.

We conducted a comparative analysis of the ASFV content in the serums of pigs with chronic and acute forms of ASF. Long viremia was observed, starting from the first clinical manifestations, and ending with the terminal stage of the disease. Viremia levels were 1.5–3.5 log lower than similar values in the acute form of ASF.

A pathological analysis of an autopsy of pig organs was also performed.

## 2. Materials and Methods

### 2.1. Animals

All the studied animals were kept at 14 farms located in the South Caucasus (38.960246, 46.595191). The first outbreak (Kovsakan 2018) was recorded in the late summer of 2018 (number of animals: 19); the second outbreak (Kovsakan 2019), in the summer of 2019 (number of animals: 19); the third outbreak (Kovsakan 2020) occurred at the end of 2019, beginning of 2020 (number of animals: 17). This article presents data from 55 pigs with unusual ASF (Arm007) variants (tentatively designated as chronic form and persistent form, which is possible with periodic reactivation).

### 2.2. Sample Collection

The Institutional Review Board/Independent Ethics Committee of the Institute of Molecular Biology of NAS RA approved the collection of biological samples (reference number IRB00004079). Biological samples were collected from August 2018 to September 2018 for Kovsakan 1 2018, from July 2019 to September 2019 for Kovsakan 2 2019, and from November 2019 to January 2020 for Kovsakan 3 2020. The studied pigs were negative for other known porcine viral diseases and vaccinated against classical swine fever. Samples from the liver, brain, bone marrow, heart, kidney, spleen, lymph nodes, and lung were kept in separate, disposable plastic containers. Clinical signs of infection were recorded daily [4]. Gross anatomical pathology characteristics were observed during routine postmortem examinations.

### 2.3. DNA Isolation and Quantitative Real-Time PCR (p72)

In order to determine ASFV gene expressions in different organs of pigs, first, total viral RNA/DNA was isolated (HiGene™ Viral RNA/DNA Prep Kit (BIOFACT Daejeon, Republic of Korea)). Quantitative real-time PCR was carried out using SYBR green methods on an Eco Illumina Real-Time PCR System device (Illumina Inc. San Diego, CA, USA) [18,19]. Each reaction mixture (20 μL) composition was as follows: 4 μL 5 × HOT FIREPol^®^ EvaGreen^®^ qPCR Mix Plus (ROX) (Solis BioDyne Tartu, Estonia), 0.2 μL of each specific primer, 4 μL template DNA/cDNA, and 11.6 μL ddH20. The thermal profile was set as follows: Polymerase activation: 95 °C for 12 min, 40 cycles: 95 °C for 15 s, 52 °C for 30 s, and 72 °C for 30 s. Standard curves were generated using serial 10-fold dilutions of viral DNA. The fluorescence threshold (Ct) was calculated using the ECO-Illumina system software v5.0. Primers used for amplification were designed and ordered from Integrated DNA Technology-IDT (https://www.idtdna.com/pagesas) (accessed on 5 February 2019) [20].

The primers and fluorescent-labeled probe used were as follows:

ASFV B646L gene:

Fluorescent probe—6-FAM/TAMRA

Sequence 1—TGC TCA TGG TAT CAA TCT TAT CG

Sequence 2—CCA CTG GGT TGG TAT TCC TC

Sequence 3—/56-FAM/TTC CAT CAA AGT TCT GCA GCT CTT/36-TAMSp/

β-actin gene:

Fluorescent probe—TET/ZEN/IBFQ

Sequence 1—CTC GAT CAT GAA GTG CGA CGT

Sequence 2—GTG ATC TCC TTC TGC ATC CTG TC

Sequence 3—/5TET/AT CAG GAA G/Zen/G ACC TCT ACG CCA ACA CGG/3IABkFQ/

The β-actin gene was used as a housekeeping gene.

To align the cDNA plots and ASFV infection titers, Cq values were rescaled after comparison with viral genome copies and modified in absolute amounts along the y-axis for better visualization. To evaluate the profile of the ASFV replication efficiency, the genes with different temporal expression patterns were identified [21,22].

### 2.4. Hemadsorption Assay

A hemadsorption assay (HAD) was performed and expressed in log 10 hemadsorption units (HADU50/mL) [23].

### 2.5. Tissue Samples

Liver, kidney, and lung samples were preserved for 24 h in 10% buffered formalin solution (pH 7.2). Following fixation, the specimens underwent a progressive phase of alcohol dehydration, followed by a xylol wash, and a routine process for embedding in paraffin wax in preparation for light microscopy. Wax-embedded samples were cut (Microm HM 355; 5 µm) and stained with hematoxylin and eosin in accordance with the manufacturer’s instructions (Sigma-Aldrich, Steinheim am Albuch, Germany) for structural analysis. A light microscope was used to conduct the histological examination.

### 2.6. Serum Collection and ELISA

Blood and serum were obtained via puncture of the jugular vein using a vacutainer system. Healthy porcine blood samples were taken to obtain control values. For the detection of TNF-a levels in serum, a commercial ELISA kit (#MBS745775; MyBioSource, San Diego, CA, USA) was used.

### 2.7. Cytology of Blood and Hemolymphoid Organs

Blood smears, lymph nodes, spleen, and bone marrow were prepared routinely according to [24]. In accordance with the manufacturer’s instructions (Sigma-Aldrich), slides were fixed in pure methanol and stained using modified Giemsa solution (aquare B/aquare II, eosin, and methylene blue) for cell examination. Using a light microscope set to 1250 magnification, cells were examined and counted in 100 randomly chosen fields (0.01 mm^2^). The microscopic evaluation of cells based on morphological characteristics was performed as described previously [25]. The evaluation of monocytes, monoblasts, and macrophages was based on morphologic characteristics [26].

Each print or smear was reviewed by three independent experts. Average data were provided for a minimum of 1000 cells in each preparation.

The main criteria for describing atypical lymphocytes in ASF have been described previously [27]. For atypical lymphocytes, the main criteria are altered nuclei and/or larger cells with slightly less condensed chromatin and cells with a lower nucleus:cytoplasm ratio. Also, an increased amount of nuclear DNA (hyperdiploid or polyploid nuclei) is often described.

## 3. Results

### 3.1. Virus Load

**Figure 1:**
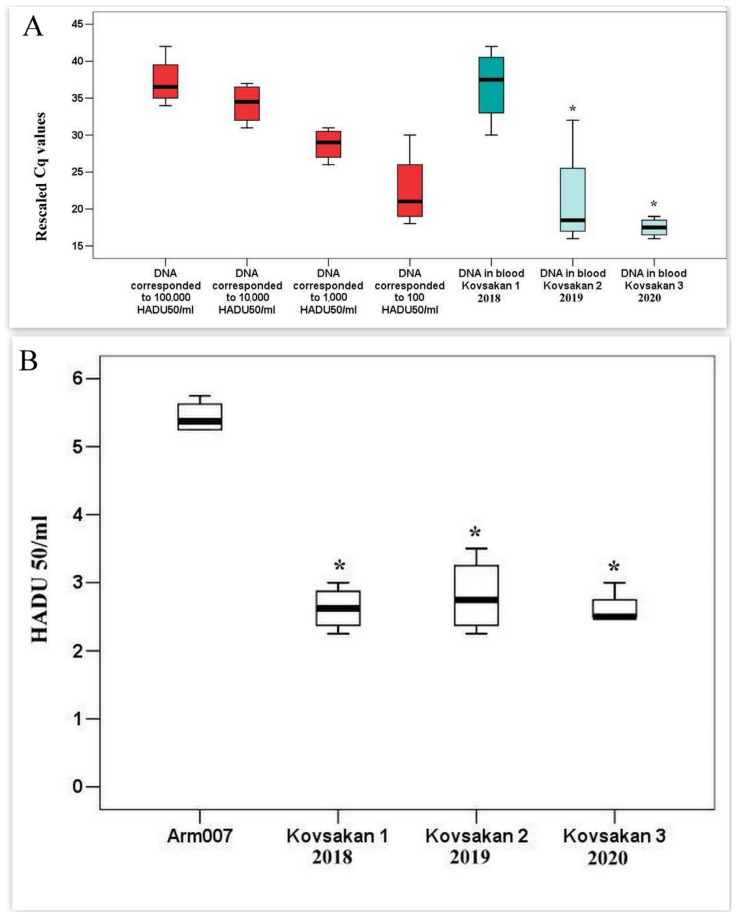
ASFV HADU and DNA levels (rescaled Ct values) in acute and chronic infected pigs’ serum. (**A**) ASFV DNA levels p72 (Ct values from real-time PCR) in the blood of infected pigs. * Significant compared to Kovsakan1 (*p* < 0.05) (**B**) HADU levels in the blood of infected pigs. * Significant compared to Arm 007 (*p* < 0.05–0.01).

The presence of ASFV DNA in the blood and spleen was confirmed via polymerase chain reaction (PCR) (Figure 1A). We contrasted the nucleotide sequences from the p72-based PCRs with those from representative samples that were previously reported (unpublished data). As predicted, the Dilijan 2011 IMB ASFV clustered within p72 genotype II. It demonstrated 100% nucleotide similarity with all compared ASFVs circulating in the Caucasus regions since 2007.

The content of the ASF virus (Kovsakan 1/2018 isolate) in the blood of sick pigs approximately corresponded to 10^5^ units of HADU when infected with the Arm07 isolate (Figure 1A). This index is approximately 0.5 decimal logarithms lower than the maximum determined during infection with Arm07 isolate. However, taking into account the fact that it was difficult to determine the maximum titers of the virus (Kovsakan 1/2018) in the blood of pigs due to the duration of the disease, it can be assumed that the number of genome copies is approximately the same when infected with Arm07 and Kovsakan 1/2018 isolates. There is a discrepancy in the quantitative indicators of the ASF virus between the RT-PCR and HAD data when analyzing the viremia of the Kovkasan1/2018 isolate (a decrease in virus content expressed in HADU is not accompanied by a decrease in copies of the gene encoding p72). This is most likely explained by the reduced HAD activity of all new virus isolates. The number of genome copies of isolates Kovsakan 2/2019 and Kovsakan 3/2020 was significantly lower and corresponded to 10^2^ HADU (Figure 1A). The titer levels of all tested virus isolates in the blood of infected pigs, when analyzed via HAD, were significantly lower than those during infection with the Arm07 isolate (approximately 2.0 decimal logarithms) (Figure 1B). In studied samples, not only the levels of HADU per ml, but also the intensity of hemadsorption were reduced. 

### 3.2. Epidemiology

All three outbreaks were identified in the South Caucasus (Kovsakan). The first outbreak (Kovsakan 1/2018) was recorded in the late summer of 2018 (August–September); the second outbreak (Kovsakan 2/2019), in summer 2019 (July–September); the third outbreak (Kovsakan 3/2020), occurred from November 2019 to January 2020.

The sources of ASF disease in all of the three outbreaks were not identified. All cases of the disease were detected in small farms (the number of animals in the farms usually ranged from 2–3 pigs to 15–20). There was a wild boar population in the region, but there were no wild boars or forest areas in the vicinity of the farms. In some of the affected farms (mostly with very few animals), the pigs were kept free-range. Most of the larger farms kept animals in barns without free range.

On the other hand, before the detection of ASF, there was a free exchange of animals and feed between farmers, which explains the duration of the outbreak and the route of infection within the rural community.

### 3.3. Clinical Manifestation

In the Kovsakan 1/2018 cases, the aged sows were affected more often than young piglets (up to 3 months old) [4]. Nineteen of them developed the disease described below (Table 1). 

The time of illness was noted from the moment the first symptoms were observed: apathy, sometimes temporary loss of appetite, and sometimes short-term fever. Among the diseased animals, 12 were 8–21 months old, and 7 were piglets aged 2–2.5 months. Weight loss and some apathy were observed in all investigated animals. Arthritis was observed in some pigs (2 animals) and also short-term attacks of fever (14 animals). Fever can be described as moderate and irregular, but is often absent. The disease was characterized by a permanent, but prolonged tendency to worsen. Petechial hemorrhages (sometimes localized only on the ears) occur at a late stage of the disease (1–4 weeks after the onset of the first symptoms), and the intensity of hemorrhages varies greatly in different animals. Clinical disease in animals infected with ASFV Kovsakan 1/2018 progressively worsened, with all animals euthanized in extremis by 35–44 days post infection. 

The time of illness was noted from the moment the first symptoms were detected: loss of appetite and rise in temperature. Among the investigated animals, 19 were 6–18 months old, and 17 were piglets aged 2–4 months. Clinical manifestations in pigs infected with Kovsakan 2/2019 (*n* = 19) and Kovsakan 3/2020 (*n* = 17) isolates were almost similar but milder. In the latter cases, sometimes clinically detectable symptoms disappeared and reappeared. It was not possible to reliably identify the causes of this phenomenon (recurrence of persistence or cure with a new infection). Some pigs have survived long enough to show signs of recovery, even to the point of gaining weight.

Table 1 represents the average life expectancy in ASFV-infected pigs (days) after the first symptoms arose until the last stage of the disease in both groups. As shown, the average life expectancy increased in a new form of ASF at least three times. In two cases, pigs survived the disease. In this case, ASF symptoms disappeared, and after 3 months, all samples from blood, urine, and stool were free from ASFV (the evaluations were performed via HAD). Other animals were euthanized at the last stage of the disease. In the chronic form of ASF, the temperature curve after the early stage of the disease shows a tendency to decrease down to normal levels, with separate short-term paroxysms. For pigs infected by the chronic strain of ASF, one of the specific characteristics of the disease was a lack or absence of skin hemorrhages, almost total absence of blood in urine and stool.

In investigations on the serum levels of TNF-alpha, pigs infected by all chronic ASFV strains were significantly lower compared with the acute form of the disease (all measurements were performed at a late stage of the disease).

### 3.4. Pathology

#### 3.4.1. Kovsakan 1/2018 

An autopsy revealed severe damage to internal organs. Severe damage was found in the vast majority of autopsy cases. This manifested presenting with single and limited hemorrhages, hemorrhages in the lungs, intestine, liver, and kidneys. In some cases, bleeding was observed in the abdominal cavity, as well as blood-stained pleural effusion. Splenomegaly with massive hemorrhages was observed in 63.6% of the pigs (12 out of 19).

#### 3.4.2. Kovsakan 2/2019 and Kovsakan 3/2020

All examined organs were edematous. There were no massive hemorrhages. In the lungs, isolated pinpoint hemorrhages present were. Splenomegaly with massive hemorrhages was observed in 13.3% of the pigs (2 out of 17).

Macroscopically, hemorrhages were detected in some lymph nodes.

### 3.5. Histopathology

#### Kovsakan 1/2018, Kovsakan 2/2019, and Kovsakan 3/2020

A histological analysis revealed isolated foci of microhemorrhages and diapedesis of erythroid tissue in lymph nodes. In lungs, isolated pinpoint hemorrhages were present.

**Figure 2:**
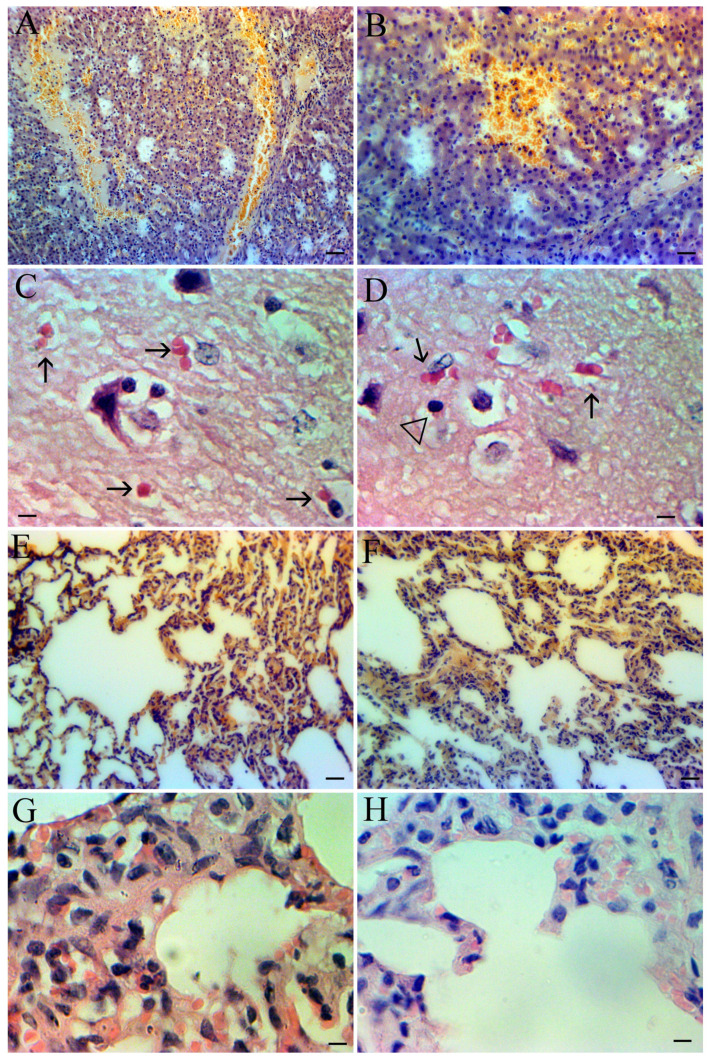
(**A**) Diapedesis and local hemorrhages in the liver at the late stage of the chronic form of ASF. Scale bar 100 µm. (**B**) Local hemorrhage in the liver at the late stage of the chronic form of ASF. Scale bar 50 µm. (**C**) Mild diapedesis in brain tissue. Erythrocytes in brain (arrowed). Scale bar 10 µm. (**D**) Mild diapedesis in brain tissue. Erythrocytes in brain (arrowed), and lymphoid cell (triangle). Scale bar 10 µm. (**E**) Mixed diapedesis in lung tissue at the late stage of the chronic form of ASF Scale bar 50 µm. (**F**) Mixed diapedesis and thickness of the alveolar wall at the late stage of the chronic form of ASF. Scale bar 50 µm. (**G**) Thickening of the interalveolar septa due to erythrodiapedesis and leukodiapedesis. Kovsakan2019. Scale bar 10 µm (**H**) Thickening of the interalveolar septa due to erythrodiapedesis and leukodiapedesis. Kovsakan2020. Scale bar 10 µm.

The histological structure of the kidneys was preserved, with a mild proliferation of mesangial cells (similar to chronic mesangioproliferative glomerulonephritis). The epithelium of the proximal convoluted and straight tubules was preserved with protein-containing urine in the lumen (Figure 2A,B).

The general lobular structure of the liver was preserved, and there was congestion in the vessels of the portal tract and sinusoids, especially in the central part of the lobules and central veins, possibly due to stagnation of blood in the systemic veins, possibly due to heart failure. Portal fields were slightly enlarged. There were activated Kupffer cells, single lymphocytes, and neutrophils (segmented and bend), especially in the central part of the lobules around necrotic hepatocytes. Diapedesis hemorrhages and necrotic changes were detected in all areas (Figure 2A,B).

In the brain tissue, the extravasation of individual cells (mainly erythrocytes and leukocytes) was observed (Figure 2C,D).

There was pronounced venous congestion in the lungs with foci of hemorrhage, interalveolar septa were thickened due to venous congestion and connective tissue growth, and alveoli were mostly empty (Figure 2E,F). Interalveolar septa were thickened via erythrodiapedesis and leukodiapedesis (Figure 2G,H).

Overall, the histopathological findings resemble those of the chronic forms of ASF with mild pathogenesis.

### 3.6. Immunopathology

#### Cellular Immune Responses Associated with Chronic ASF

**Figure 3:**
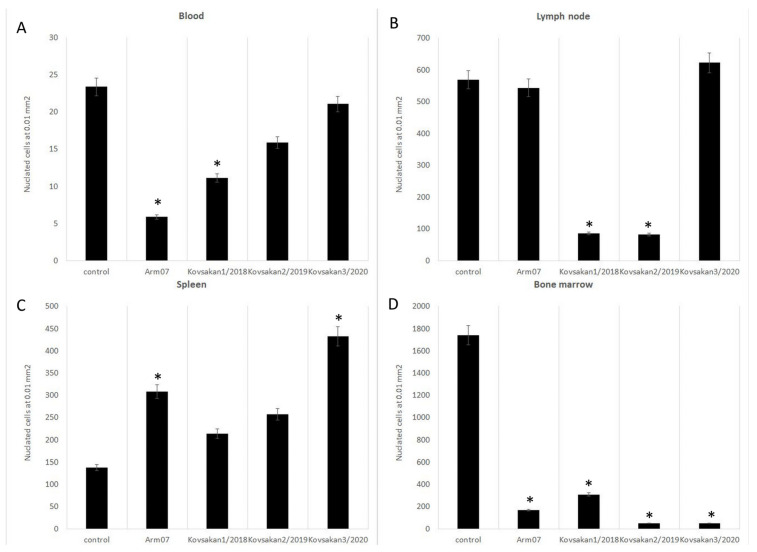
Nucleated cell amount in the blood, lymph nodes, spleen, and bone marrow at late stages of Kovsakan1/2018, Kovsakan2/2019, and Kovsakan3/2020. (**A**) Nucleated cell amount in the blood. (**B**) Nucleated cell amount in lymph nodes. (**C**) Nucleated cell amount in the spleen. (**D**) Nucleated cell amount in the bone marrow. * Significant compared to control (*p* < 0.05–0.01).

It was previously shown that the number of leukocytes in the blood of ASFV-infected pigs significantly decreased at the last stages of acute ASF, and this was also observed in the chronic form of ASF. The total white blood cell count in the Kovsakan 1/2018 ASF form was slightly (insignificantly) below the cutoff for the established normal range (Figure 3A), but significantly higher compared with the acute form (Arm007). In Kovsakan 2/2019 and particularly in Kovsakan3/2020, the numbers of nucleated cells in blood were significantly higher compared with those in the acute form (Arm007) and healthy pigs; the data of Kovsakan 3/2020 isolate correspond to the data of the norm (and Arm007). 

In lymph nodes, the cell counts for Kovsakan1/2018 and Kovsakan2/2019 were significantly lower (Figure 3B) compared with that of the acute form (Arm007). A sphenogram (Figure 3C) in pigs with Kovsakan3/2020 revealed that the number of nucleated cells in blood was significantly higher compared with that of the acute form (Arm007). A myelogram (Figure 3D) reveals that the nucleated cell numbers for Kovsakan2/2019 and Kovsakan3/2020 were significantly lower (Figure 3D) compared with those of the acute form (Arm007).

Marked neutropenia (mature forms) were observed in the late stages of Kovsakan2/2019 and Kovsakan3/2020, but not Kovsakan1/2018 (Table 2). The percentage of lymphoid cells in the blood of pigs with the Kovsakan3/2020 form almost corresponds to the control values; however, at the same time, the content of monocytes sharply decreases.

As follows from Table 2, with Kovsakan1/2018, a more prominent shift is observed to the left in all WBC populations of peripheral blood compared with acute ASF (lymphoid, myeloid populations). This phenomenon occurs in all described forms of the disease, but the most obvious is Kovsakan1/2018. Also, significant amounts of destroyed cells arise. However, the smudge cell numbers at terminal stages of all forms of ASF, except in cases of Kovsakan2/2019 and Kovsakan3/2020, were more prominent.

As follows from Table 3, upon ASF (Arm07) infection, a severe lymphopenia in the BM was detected in the late phase of infection. Isolates Kovsakan1/2018 and Kovsakan2/2020 also showed reductions in lymphocytes, however less prominent. Isolate Kovsakan3/2020 showed a reverse tendency to increase the percentage of lymphoid cells. Very prominent decreases in monocytes and monoblasts occurred in all new isolates of ASFV. The content of nucleated erythroid cells corresponded to the control values and was significantly lower compared to the acute form caused by the Arm007 isolate (Table 3), and Kovsakan3/2020 exhibited a significantly increased amount of macrophages, sometimes with a pathologically activated phenotype.

In BM, a sharp decrease in the content of all nuclear cells is noticeable in all forms of ASF. The most pronounced pancytopenia was observed in Kovsakan3/2020 when the BM can be assessed as almost empty. The main feature of the disease caused by isolate Kovsakan3/2020 is a significantly increased number of macrophages, almost all of them with pathologically activated phenotypes. Similar macrophages in infection with other forms of ASF are also found, but much less frequently. In acute ASF, the number of lymphocytes significantly decreased, and the same occurred in Kovsakan1/2018 and 2/2019. The numbers of monocytes and monoblasts, as well as mature neutrophils, decreased in all investigated isolates observed at 3 dpi (Table 3). Compared with those in healthy pigs, immature myeloid cells increased in Kovsakan1/2018 and partially in Kovsakan2/2019, but decreased in Kovsakan3/2020. The number of nucleated erythroid cells significantly decreases with Kovsakan1/2018 infection, is significantly higher than in healthy pigs after infection with Kovsakan2/2019, and approximately corresponds to the norm in Kovsakan3/2020.

As follows from Table 3, as terminal stages of chronic ASF take place, a significant decrease in all forms of elytroid populations in the BM was observed, compared not only with the control but also with acute ASF. Also, BM smears showed a more prominent shift to the left in the myeloid population compared with terminal stages of acute ASF. This also describes the proliferation of mature macrophages in BM tissue with a significant increase in the macrophage number compared with corresponding indices at terminal stages of acute ASF. There were no significant changes in other cell populations.

The general trend of changes in the population composition of spleen cells (Table 4) is reduced to a decrease in the content of early cells (lymphoblasts, monoblasts, myeloid cells) with an increase in the content of lymphocytes.

During the late stages of the disease, ASF (Arm07) infection in lymph nodes also caused a twofold reduction in lymphocyte counts, which was accompanied by an increase in monocyte counts (lymphopenia) (Table 5). Lymphopenia was most pronounced in Kovsakan1/2018; Kovsakan2/2019 has an intermediate position; and Kovsakan3/2020, according to the content of lymphocytes, corresponded to the norm. According to the content of nucleated erythroid cells, Kovsakan1/2018 and 2/2019 correspond to the pathology caused by Arm, and Kovsakan3/2020 practically corresponds to the norm.

**Figure 4:**
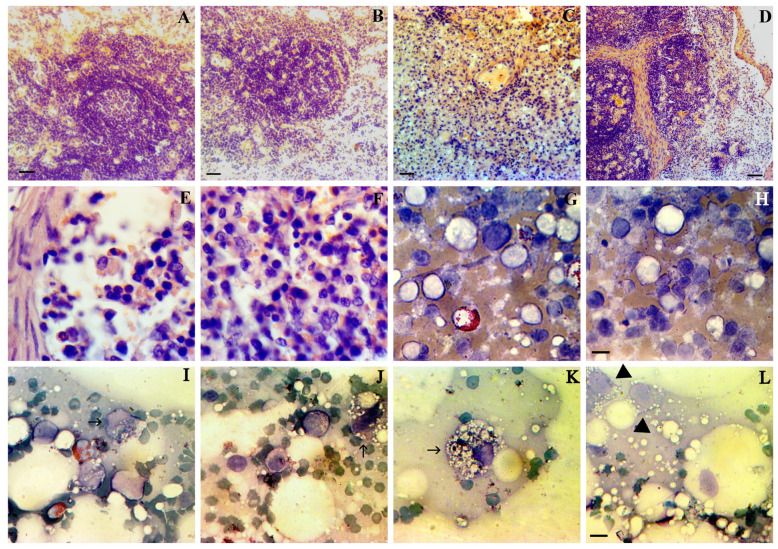
Pathology of immune organs in the late stages of the Kovsakan3/2020 form of ASFV. (**A**) Lymph node. Preserved structure and cellularity. Small but multiple foci of erythrocyte infiltration. Staining by hematoxylin eosin. Scale bar 50 µm. (**B**) Lymph node. Preserved structure and cellularity. Small but multiple foci of erythrocyte infiltration. Staining by hematoxylin eosin. Scale bar 50 µm. (**C**) Lymph node. Severe diapedesis. Staining by hematoxylin eosin. Scale bar 50 µm. (**D**) Lymph node. Preserved structure of the lymph node and absence of hemorrhages. Staining by hematoxylin eosin. Scale bar 100 µm. (**E**) Spleen, follicle local reduction in nucleated cells. Staining by hematoxylin eosin. The scale bar for E-H is 10 µm. (**F**) Spleen, almost intact follicle with mild diapedesis. Staining by hematoxylin eosin. (**G**) Spleen, reduction in nucleated cells. Staining by Giemsa. (**H**) Spleen, lymphocytosis. Staining by Giemsa. (**I**) Bone marrow smear. Pancytopenia. Hyperactive macrophage (arrowed). Staining by Giemsa. The scale bar for I-L is 10 µm. (**J**) Bone marrow smear. Pancytopenia. Hyperactive macrophage (arrowed). Myeloid cells. Staining by Giemsa. (**K**) Bone marrow smear. Pancytopenia. Hyperactive giant macrophage (arrowed). Staining by Giemsa. (**L**) Early blast cells with vacuolized cytoplasm (triangles), erythroblastic islet with minimal erythroblasts. Staining by Giemsa.

In Figure 4, we present the histopathology of immune organs with the Kovsakan3/2020 form of ASFV. In the lymph nodes, with preserved structure and cellularity, there is a slight noticeable erythrocyte infiltration (Figure 4A,B), a diapedesis (Figure 4C); however, usually, hemorrhages are not described (Figure 4D). Spleen follicles, either intact or demonstrated, observed a local reduction in nucleated cells (Figure 4E–H). In bone marrow, sever pancytopenia was detected, with infiltration of macrophages, sometimes with a hyperactive phenotype (Figure 4I,J). Some macrophages, in addition to vacuolization, were sharply increased in size (Figure 4I,K). A population of early unidentifiable cells was also noted (Figure 4L). Erythroblastic islets were preserved, but the number of erythroblasts in them was minimal (Figure 4L).

## 4. Discussion

In Eurasia, the emergence of several variants of the ASF virus has been shown to occur within a short period of time, and the virus has been shown to differ in virulence [8]. Sometimes, the change in virulence of a virus can be explained as a result of major genetic changes [28], and sometimes, the explanation of low virulence requires further investigation [29]. Three isolates of the ASF virus with moderate pathological manifestations and reduced mortality have been identified in the South Caucasus in 2018–2020, differing from each other in different degrees of attenuation.

The new ASF isolates cause an unusual clinical picture—described isolates differ from the usual subacute form by a reduced ability of hemadsorption and a reduced frequency and intensity of hemorrhages. A less pronounced lesion of the blood coagulation system is also shown, compared with acute ASF [30]. The causes of subacute and chronic forms may be penetration into the primary ASF zone of spread of new types of viruses with lower virulence, or a change in the virus with the appearance of new, less virulent mutant strains. So, along with the primary highly virulent isolate identified in the Republic of Armenia in 2007, we can conclude that at the end of 2011, a moderately virulent isolate for domestic pigs appeared [4].

The main aspect of the attenuation of the described isolates of the ASF virus is the long-term carrying of the virus with viremia [31]. At the same time, the level of the virus in the blood and tissue of infected animals is significantly lower than that when infected with a highly pathogenic strain (Arm07). For the attenuation of the virus, either increased sensitivity to the antiviral defense of the host is required, or a decrease in the replication activity of the virus, or changes in the virus genome [8].

Generally, a lower virulence of ASFV is associated with genomic changes present in attenuated ASFV strains and differences in the immune response of infected animals [32]; a combination of both factors is also possible. The decrease in virulence can be explained by the fact that pigs may have been previously exposed to ASF virus and survived. On the one hand, this assumption is supported by the fact that several outbreaks of ASF were recorded in a relatively small region over several years. However, most of the affected pigs were between several weeks and several months old and would not have had enough time to become reinfected with ASF. Spontaneous abortions in pregnant pigs were not observed on the farms investigated. Viremia was not detected in healthy pigs from neighboring farms or in the majority of healthy pigs (either via rtPSR or HAD). These data reduce (but do not exclude) the likelihood of reinfection with ASF virus.

The decrease in virus virulence can also be explained by the transfer of antibodies to the ASF virus in the mothers’ milk. It is known that when antibodies are transferred via milk, virus titers into the blood of piglets, the duration of viremia decreases, and the clinical manifestation becomes much less pronounced [33]. This is explained by the protective effect of passively acquired antibodies [34]. Therefore, this aspect cannot be ignored, especially as some sows were more severely affected by ASF than the piglets.

From our point of view, the most likely explanation is a change in the genome of the virus, due to the evolution characteristic of viruses. From studying the evolution of viruses, we know that genomic changes will almost inevitably reduce the pathogenicity of viruses given enough time [35]. Although DNA viruses have a significantly lower rate of change in the genome, their variability far exceeds that of cellular organisms. Draft sequences of some genes shown modest mutations compared to ASFV Georgia 2007 (Figure S1 MGF 360-2L gene; Figure S2 G1340L gene; Figure S3 MGF360-11L gene; Figure S4 MGF360-10L gene) [36,37] The emergence of new, less virulent ASF virus mutants undoubtedly has an important role in the survival of the virus, increasing the possibilities of its transmission. It is commonly recognized that DNA viruses frequently cause chronic, invisible, long-lasting illnesses [38]. The changes in the clinical manifestations of the unusual ASF form described in our article support this view. The changes we detected in the clinical manifestations of the disease confirm this point of view.

One of the most impressive manifestations of the pathology of partially attenuated ASFV isolates is a very pronounced pancytopenia accompanied by the devastation of all hematopoietic organs, primarily the bone marrow. Numerous viruses, both human and animal, can result in severe bone marrow depression. These viruses include the human immunodeficiency virus, dengue virus, parvovirus B19, Epstein–Barr virus, cytomegalovirus, and feline leukemia and panleukopenia viruses [39]. The authors state that pancytopenia’s clinical manifestation can result from a number of pathogenic pathways. A number of viruses have the potential to directly harm hematopoietic progenitors, expose the bone marrow to immune-mediated destruction, or cause the stromal microenvironment’s vital nurturing elements to be lost. We tend to consider the second postulate as the most probable. Previously, we identified the occurrence of hemophagocytic lymphohistiocytosis in the acute form of ASF and provided its criteria in pigs [40], which allows us to assume similar pathogenesis and lethal forms when infected with partially attenuated ASF virus isolates.

It should also be noted that in the blood and lymph nodes, the total white blood cell content does not differ from that of healthy pigs (unlike in the acute form of ASF and Kovsakan2018 infection); therefore, compensatory mechanisms of bone marrow depletion should not be excluded. This is also supported by splenic hyperplasia with the possible development of compensatory extramedullary hematopoiesis [41].

Kovsakan2/2019 and Kovsakan3/2020 isolates are very close to each other in terms of clinical manifestations and the amount of ASFV in the blood of the pigs but differ in terms of pathological and laboratory parameters. We do not rule out that these isolates are varieties of the same virus or that the above differences are a reflection of different stages of the disease. The latter is quite likely, since the disease is characterized by a very blurred nature, and it is difficult to determine whether the animals are in the stage of manifestation or of convalescence of the disease.

## 5. Conclusions

The identified new virus isolates resemble a strongly prolonged form of persistent ASF with reduced mortality. Therefore, the most important characteristics of the natural attenuation of the ASF virus should be considered a reduction in the infectious titers of the virus in the blood and a change in the pathogenesis of the disease.

The direct mechanisms of virus attenuation have not been reliably identified; however, the pathogenetic reasons are a pronounced decrease in the manifestations of the symptoms of the disease, which allows the bodies of pigs to develop compensatory mechanisms.

The undoubted reason for attenuation from the perspective of the virus is a pronounced prolongation of the viremia.

## Figures and Tables

**Table 1:** Clinical characteristics of naturally attenuated forms of African swine fever.

	Arm07	Kovsakan2018	Kovsakan2019 and Kovsakan2020
	Late Stage	Late Stage	Late Stage
**Permanent loss of appetite (%)**	100	100	47
**Skin hemorrhages**	Severe (on ears, abdomen, etc.)	Mild (predominantly on ears)	Mild (predominantly on ears)
**Internal bleeding**	Severe	Mild	Mild, sometimes absent
**Lethality (%)**	100	12 out of 19 (63.6%)	4 out of 17 (26.7%) *
**Average life expectancy (day)**	4–7	25–35 *	48 and more *

* significant compared with Arm07 (*p* < 0.05–*p* < 0.01).

**Table 2:** Blood cell populations compared in the late stages of Arm07 and Kovsakan1–3 infections.

Blood		Arm07	Kovsakan1/2018	Kovsakan2/2019	Kovsakan3/2020
Cells	Control	Late Stage	Late Stage		
**Erythroblasts**	0	3.1 ± 0.3	8 ± 0.9 *	6.2 ± 0.9	4.1 ± 0.5
**Lymphoblast**	0	2.8 ± 0.3	5 ± 0.9 *	6.3 ± 1.1	2.2 ± 0.1
**Lymphocyte**	56.0 ± 4.7	44 ± 6.1	24 ± 3.6 *	32.9 ± 4.4	53.7 ± 6.9
**Monoblast**	0.5 ± 0.1	0.2 ± 0.01	5 ± 0.7 *	1.0 ± 0.3	0.8 ± 0.2
**Monocyte**	7.7 ± 1.3	7.4 ± 0.8	8 ± 1.1	3.3 ± 0.5	1.5 ± 0.1 *
**Myeloid**	0	0.1 ± 0.01	2 ± 0.4 *	0.8 ± 0.2	1.3 ± 0.3
**Metamielocyte**	0.1 ± 0.02	7.3 ± 1.0	12 ± 2.1 **	2.1 ± 0.5	5.8 ± 1.4 **
**Band**	7.7 ± 1.0	5.6 ± 0.7	13 ± 0.9 *	9.9 ± 1.4	6.8 ± 0.3 *
**Segment**	23.1 ± 3.3	1.9 ± 0.3	5 ± 0.8 *	16.1 ± 3.4	7.7 ± 1.2 *
**Pathol neutrophil**	0	2.1 ± 0.5	9 ± 1.1 *	5.6 ± 2.1	2.4 ± 0.6 *
**Eosinophil**	4.9 ± 2.1	1.9 ± 0.4	2 ± 0.3	2.8 ± 0.8	3.5 ± 0.9
**Basophil**	0.2 ± 0.1	0.4 ± 0.1	>0.1	>0.1	>0.1
**Smudge cells (remnants of cells) without clear cytoplasmic borders**	0	22.7 ± 4.7	6 ± 1.2 *	12.9 ± 4.5 *	10.1 ± 3.0 *

* significant compared with Arm07 (*p* < 0.05–*p* < 0.01). ** tendency (*p* < 0.1).

**Table 3:** Bone marrow cell populations compared in the late stages of Arm07 and Kovsakan1–3 infections.

Bone Marrow		Arm07	Kovsakan1/2018	Kovsakan2/2019	Kovsakan3/2020
Cells	Control	Late Stage	Late Stage	Late Stage	Late Stage
**Nucleated erythroid cells**	31.9 ± 4.4	32.1 ± 2.7	7.7 ± 0.9 *	54 ± 9.5 *	28 ± 4.3
**Lymphoblasts**	6.5 ± 0.8	8.1 ± 1.2	7.1 ± 1.3	2 ± 0.4 *	10 ± 0.5
**Lymphocytes**	20.6 ± 1.2	14.6 ± 2.2	23.4 ± 3.0 *	26 ± 3.8	41 ± 7.6 *
**Monoblasts**	4.2 ± 0.5	7 ± 0.9	1.0 ± 0.2	1.0 ± 0.5	2.0 ± 0.4
**Monocytes**	3.2 ± 0.8	4.1 ± 0.7	2.5 ± 0.7	0.001	0.001
**Myeloid cells**	2.7 ± 0.6	3 ± 1.0	7.6 ± 1.3 *	5.1 ± 0.7	3.0 ± 0.1
**Metamielocytes**	6.3 ± 0.9	3 ± 0.5	9.2 ± 1.1	3.0 ± 0.9	3.1 ± 1.1
**Band neutrophils**	10.6 ± 2.1	7.8 ± 0.9	20.3 ± 2.8 *	0.9 ± 0.3 *	2.8 ± 0.5 *
**Segment neutrophils**	4.1 ± 0.9	3.8 ± 0.9	1.5 ± 0.2	2.1 ± 0.6	0.9 ± 0.4 *
**Pathol neutrophils**	0	0.4 ± 0.1	1.0 ± 0.1	0	0
**Eosinophils**	8.5 ± 0.8	7.1 ± 0.9	6.1 ± 0.6	3.7 ± 0.8	3.9 ± 1.1 *
**Basophils**	1.4 ± 0.1	2.4 ± 0.2	0.5 ± 0.1 *	0.001	0
**Macrophages**	0.1 ± 0.04	1.3 ± 0.1	5.1 ± 0.9 *	1.1 ± 0.05	3.9 ± 0.6 *
**Atypical lymphocytes**	0	4.4 ± 0.3	3.0 ± 0.5	0	0
**Smudge cells (remnants of cells) without clear cytoplasmic borders**	0	1 ± 0.4	4.1 ± 0.9 *	1.9 ± 0.3	1.1 ± 0.2

* significant compared with Arm07 (*p* < 0.05–*p* < 0.01).

**Table 4:** Spleen cell populations compared in the late stages of Arm07 and Kovsakan1–3 infections.

Spleen		Arm07	Kovsakan1/2018	Kovsakan2/2019	Kovsakan3/2020
Cells	Control	Late Stage	Late Stage	Late Stage	Late Stage
**Nucleated erythroid cells**	5.5 ± 1.6	16.5 ± 2.8	5.0 ± 2.0	5.9 ± 1.1	5.8 ± 1.9
**Lymphoblasts**	5.7 ± 1.3	4.4 ± 1.6	4.1 ± 1.2	4.4 ± 1.0	1.9 ± 0.8 *
**Lymphocytes**	60.4 ± 6.3	26.1 ± 4.9	44.3 ± 4.8 *	47.3 ± 5.6	82.6 ± 9.1 *
**Monoblasts**	3.1 ± 0.9	2.4 ± 1.0	0.5 ± 0.1 *	0.5 ± 0.6	0.4 ± 0.3 *
**Monocytes**	7.0 ± 1.2	2.2 ± 0.3	0.3 ± 0.1 *	0.3 ± 0.1 *	0.2 ± 0.1 *
**Myeloid cells**	1.0 ± 0.5	10.5 ± 2.6	8.2 ± 2.3	9.2 ± 1.8	1.3 ± 0.4
**Metamielocytes**	3.1 ± 0.8	4.8 ± 1.6	5.1 ± 1.1	5.1 ± 1.2	1.0 ± 0.1
**Band neutrophils**	4.3 ± 0.7	7.1 ± 1.8	1.2 ± 0.2 *	1.2 ± 0.5 *	1.3 ± 0.1 *
**Segment neutrophils**	4.0 ± 0.7	6.5 ± 2.2	0.8 ± 0.2 *	0.8 ± 0.1 *	1.3 ± 0.1 *
**Pathol neutrophils**	0	0.1 ± 0.1	0.1 ± 0.1	0.1 ± 0.1	0.05 ± 0.1
**Eosinophils**	4.7 ± 1.0	11.0 ± 2.8	18.1 ± 3.9	3.3 ± 1.0 *	1.3 ± 0.2 *
**Basophils**	1.2 ± 0.6	1.1 ± 0.3	0.1 ± 0.1	0.0 ± 0.0	0.01 ± 0.01
**Macrophages**	0.1 ± 0.1	0.2 ± 0.1	0.3 ± 0.1	0.3 ± 0.1	0.8 ± 0.1
**Atypical lymphocytes**	0	3.0 ± 1.0	0.7 ± 0.2 *	21.6 ± 2.8 *	1.9 ± 0.7
**Smudge cells (remnants of cells) without clear cytoplasmic borders**	0.1 ± 0.1	4.5 ± 2.2	11.2 ± 3.0	4.4 ± 1.3	5.8 ± 1.7

* significant compared with Arm07 (*p* < 0.05–*p* < 0.01).

**Table 5:** Lymph node cell populations compared in the late stages of Arm07 and Kovsakan1–3 infections.

Lymph Node		Arm07	Kovsakan1/2018	Kovsakan2/2019	Kovsakan3/2020
Cells	Control	Late Stage	Late Stage		
**Nucleated erythroid cells**	1.4 ± 0.1	20.7 ± 3.4	17.9 ± 3.3	17.6 ± 1.7	0.5 ± 0.4 *
**Lymphoblasts**	1.9 ± 0.4	1.0 ± 0.7	8.1 ± 2.4 *	8.1 ± 1.1 *	1.3 ± 1.0
**Lymphocytes**	83.0 ± 5.1	38.5 ± 5.9	49.7 ± 3.7 *	60.7 ± 5.2 *	92.4 ± 10.7 *
**Monoblasts**	1.0 ± 0.2	0.2 ± 0.1	1.4 ± 0.3 *	1.4 ± 0.4 *	0.3 ± 0.1
**Monocytes**	1.1 ± 0.2	5.0 ± 1.3	0.3 ± 0.1 *	0.3 ± 0.1 *	0.3 ± 0.1 *
**Myeloid cells**	4.3 ± 1.1	18.5 ± 3.4	3.1 ± 0.4 *	3.1 ± 0.5 *	0.0
**Metamielocytes**	2.5 ± 1.0	3.7 ± 1.5	5.1 ± 0.9	0.1 ± 0.1 *	0.0
**Band neutrophils**	1.2 ± 0.5	3.9 ± 2.6	0.1 ± 0.1 *	0.2 ± 0.1 *	0.1 ± 0.1 *
**Segment neutrophils**	0.8 ± 0.4	1.2 ± 0.8	0.1 ± 0.1 *	0.1 ± 0.1 *	0.9 ± 0.2
**Pathol neutrophils**	0	0.1 ± 0.1	0.1 ± 0.01	0.1 ± 0.1	0.0
**Eosinophils**	2.2 ± 0.6	2.0 ± 0.9	1.7 ± 0.4	1.7 ± 0.4	0.3 ± 0.1 *
**Basophils**	0.6 ± 0.1	0.2 ± 0.1	0	0.0	0.1 ± 0.1
**Macrophages**	0	0.1 ± 0.1	1.0 ± 0.2	1.0 ± 0.2	0.7 ± 0.2
**Atypical lymphocytes**	0	1.0 ± 0.5	0.2 ± 0.1	5.7 ± 0.8 *	3.1 ± 1.1 *
**Smudge cells (remnants of cells) without clear cytoplasmic borders**	0.1 ± 0.1	2.1 ± 0.4	11.2 ± 4.4 *	8.1 ± 1.1 *	1.3 ± 0.6

* significant compared with Arm07 (*p* < 0.05–*p* < 0.01).

## Data Availability

Data available in a publicly accessible repository.

## Supplementary Materials

The following supporting information can be downloaded at: https://www.mdpi.com/article/10.3390/pathogens13020130/s1, Figure S1: ASFV MGF 360-2L gene (Kovsakan 3 2020) sequence compared to Georgia 2007; Figure S2: ASFV G1340L gene (Kovsakan 3 2020) sequence compared to Georgia 2007; Figure S3: ASFV MGF360-11L gene (Kovsakan 3 2020) sequence compared to Georgia 2007; Figure S4: ASFV MGF360-10L gene (Kovsakan 3 2020) sequence compared to Georgia 2007.
