# Supplementary figures and images for: Pathology and Clinics of Naturally Occurring Low-Virulence Variants of African Swine Fever Emerged in Domestic Pigs in the South Caucasus
Source: Pathogens. 2024 Jan 29;13(2):130. doi: 10.3390/pathogens13020130 (PMC10893001; doi:10.3390/pathogens13020130)

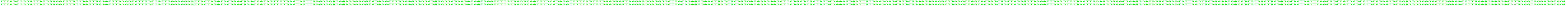

Supplement: Supplementary file 1 [file pathogens-13-00130-s001.zip › Figure S1. ASFV MGF 360-2L gene (Kovsakan 3 2020) sequence compared to Georgia 2007.jpg]

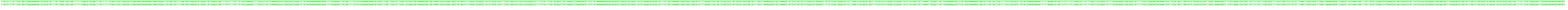

Supplement: Supplementary file 1 [file pathogens-13-00130-s001.zip › Figure S3. ASFV MGF360-11L gene (Kovsakan 3 2020) sequence compared to Georgia 2007.jpg]

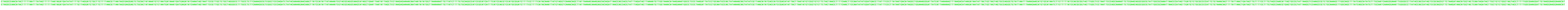

Supplement: Supplementary file 1 [file pathogens-13-00130-s001.zip › Figure S4. ASFV MGF360-10L gene (Kovsakan 3 2020) sequence compared to Georgia 2007.jpg]
